# Analysis of tether breakages during revision after failed thoracolumbar double-row vertebral body tethering

**DOI:** 10.1007/s43390-026-01345-7

**Published:** 2026-04-03

**Authors:** Jil Frank, Alice Baroncini, Mahmoud Alkharsawi, Per Trobisch

**Affiliations:** 1 Department of Orthopaedics, Trauma and Reconstructive Surgery, Uniklinik RWTH Aachen, Pauwelsstraße 30, 52074 Aachen, Germany; 2 Casa Di Cura Humanitas San Pio X, Milan, Italy; 3 Department of Spine Surgery, Eifelklinik St Brigida, Simmerath, Germany

**Keywords:** Vertebral body tethering (VBT), Tether breakage, Revision surgery, Thoracolumbar curves, Double-row implants

## Abstract

**Purpose:**

Vertebral Body Tethering (VBT) yields improving postoperative results. However, tether breakage remains a common complication, especially in thoracolumbar (TL) curves, and can negatively impact the outcome. The pathomechanism behind TL tether breakages is not well understood, yet this knowledge is essential for improving surgical planning. Therefore, the study aimed to investigate the timing and location of tether breakage in double-row (TL) revision cases.

**Material:**

This study performed a descriptive analysis of consecutively explanted double-row implants in TL VBT patients who underwent revision due to tether breakage and for whom photographic documentation was available. A > 5° increase in the interscrew angle on consecutive radiographs suggested tether breakage.

**Results:**

Data from 50 instrumented levels in ten patients were available. The UIV ranged from T9 to T11 and the LIV from L3 to L4. Revision surgery was performed, on average, 35 months after the index surgery. The study showed that the > 5° rule identified a higher percentage of segments with both tethers broken (68%) than segments with only one broken tether (54%). There was no difference in the occurrence of anterior (61%) and posterior (62%) tether breakage. The anterior tether broke in the distal segment in all ten cases, while the posterior tether broke in the distal segment in nine out of ten cases.

**Conclusion:**

This study showed that the distal segment of the lumbar spine is prone for tether breakage. Fewer breakages were observed in the thoracic segments. Additionally, no clear breakage pattern in relation to implant standing time was observed.

## Introduction

Vertebral Body Tethering (VBT), a surgical technique for scoliosis correction, has become an alternative treatment option for a well-chosen patient group [1–3]. Clinical studies have shown satisfactory and improving postoperative results. Unlike posterior spinal fusion (PSF), VBT enables scoliosis correction while preserving spinal flexibility [4, 5]. This surgical technique is suitable for skeletally immature patients and is based on the biomechanical principles of Hueter–Volkmann and Wolff’s law [6].

Nevertheless, the literature also shows that tether breakage remains one of the most common postoperative complications of this evolving technique. Breakage rates range from 30% in thoracic segments to 70% in lumbar segments [7]. Tether breakage may result in loss of correction and occasionally require revision surgery. Tetreault et al. reported ≥ 5° curve progression within two years post-breakage; 45.2% of patients experienced breakage, but only 14.5% underwent revision [8–10]. Understanding TL breakage mechanisms is critical for planning instrumentation levels and surgical timing.

Research is ongoing about the exact pathomechanism and consequences of tether breakage. Previous studies found that breakage in the first year after surgery leads to curve progression, whereas breakage after 12 months has limited clinical relevance [8]. Biomechanical studies have analyzed the prevailing tether tension forces during various physiological movements using a multi-body simulation model. In particular, increased tether tension forces were observed during lateral bending to the untethered side, which may compromise the structural integrity of the tether [11]. In addition, VBT combined with apical fusion has been shown to reduce tether forces not only within the fused segment but also in adjacent segments, providing a possible explanation for the clinically observed reduction in tether breakage [12, 13].

Since the tether is radiolucent, imaging can only suggest a possible tether breakage, and confirmation can only be obtained when implants are explanted [1]. Trobisch et al. described a low sensitivity of the > 5° rule [14]. Hence, little is known about the tether breakage patterns with respect to location and time. A recently published study analyzed in situ breakage in relation to the instrumented vertebrae. However, the patient population was heterogeneous and included thoracic, TL, as well as single- and double-row tethers [15].

This study aimed to analyze tether breakages following failed double-row thoracolumbar VBT, focusing on the location of breakage and the timing of revision surgery. A deeper understanding of the pathomechanics underlying TL tether breakages is essential for improving surgical planning, particularly with regard to determining optimal instrumentation levels and surgical timing.

## Methods

This was a retrospective single-institution study on prospectively collected data. Ethical approval was obtained from the local ethics committee.

### Patients’ recruitment

**Fig. 1:**
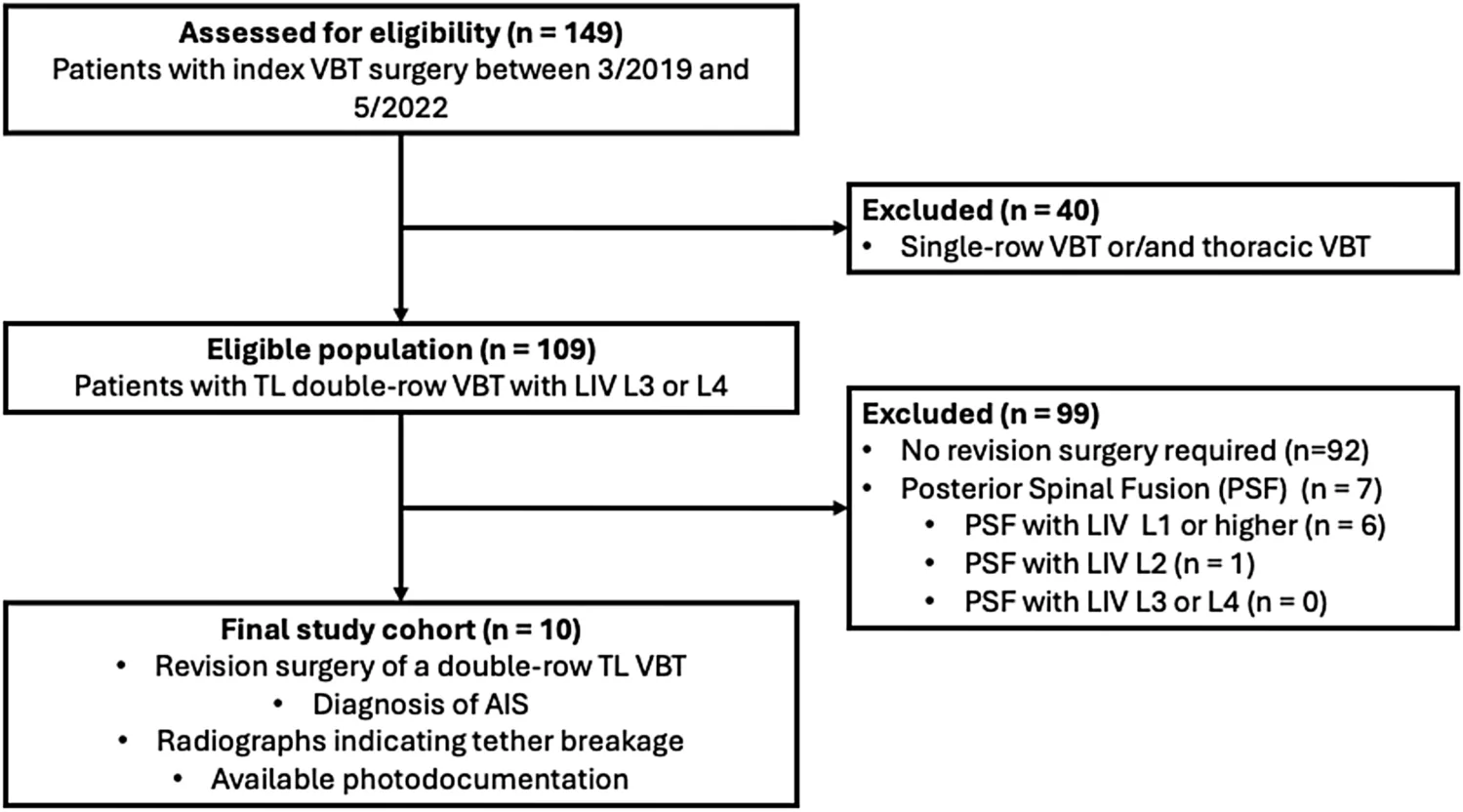
Flowchart showing the inclusion criteria and patient selection for the analyzed cohort of TL double-row revision surgery

The study was conducted on consecutively operated patients with double-row TL VBT who underwent revision surgery for tether breakage between August 2022 and December 2024 at the same institution and for whom photographic documentation was available. **(**Fig. 1**)** The index and revision surgeries were performed at the same institution that introduced double-row VBT in 2019. All the tethers used for index surgery were 4 mm. The inclusion criteria were as follows: a diagnosis of AIS; a revision of thoracolumbar double-row VBT; radiographs indicating a suspected tether breakage; an available photodocumentation; and radiographs taken before revision surgery. Patients with single-tether TL instrumentation were excluded from the study. Tether breakage was suspected radiographically when a > 5° increase in angulation between two screws was observed on two consecutive radiographs [15]. Indications for revision surgery were defined as a significant loss of correction > 40°, the radiographic suspicion of tether breakage, and/or the development of pain. In addition, the development of coronal decompensation and the patient’s dissatisfaction were used as indications for revision surgery. [1, 14, 16].

### Data collection

Baseline demographic data including gender and age were collected. To determine skeletal maturity, Risser and Sanders Stages were determined for each patient. Anteroposterior and lateral standing full spine radiographs were obtained pre- and postoperatively. Thoracic and lumbar Cobb angles as well as thoracic and lumbar bending Cobb angles were determined preoperatively. Postoperative radiographs were obtained at first erect, 12 months, 24 months after index surgery. Additionally, radiographs were obtained before revision surgery. The thoracic and lumbar Cobb angles were measured and monitored throughout the follow-up period.

### Analysis of time and location of tether breakages (TB)

Two authors, who were not involved in the surgeries or postoperative follow-ups, assessed the photographs of the explanted tethers. The surgeon annotated the anterior and posterior tethers with their respective positions and instrumentation levels. Pictures depict screw fixation sites (arrows), corresponding vertebral bodies, and tether rupture points, enabling precise postoperative interpretation.

### Surgical technique

Revisions were performed using a surgical technique that was resembling to that used for the index surgeries. Patients either received a re-tethering when still immature or a partial anterior fusion with intervertebral cages in combination with a tether exchange.

Surgery was performed with the patient in the lateral decubitus position under general anesthesia with single-lung ventilation. In all cases, the same skin incision was reused with no requirement for extension. Typically, the surgical approach for L1 and above (including the L1/2 intervertebral disc) was carried out through intercostal transpleural access. Pleural or pulmonary scarring was not observed in either case when UIV was T10 and only light scarring that could be solved with blunt dissection when UIV was T9. L2 to L4 were operated with a direct lateral, retroperitoneal, anterior-to-psoas approach. Significant scarring was not observed. However, we recommend to dissect between the psoas fascia and the psoas muscle to get access to the anterior border of the psoas and retract the muscle posterior. Dissecting below the fascia prevents potential injury to the ureter [16].

### Statistical analysis

The time-to-breakage and the location of the breakage (level, anterior or posterior tether) were investigated. Descriptive statistics were performed for all analyzed data. Continuous data were expressed as mean ± standard deviation, while qualitative data were expressed as rates and percentages.

## Results

### Patients’ recruitment

Ten patients met the inclusion criteria and were included in the study.

### Baseline data

**Fig. 2:**
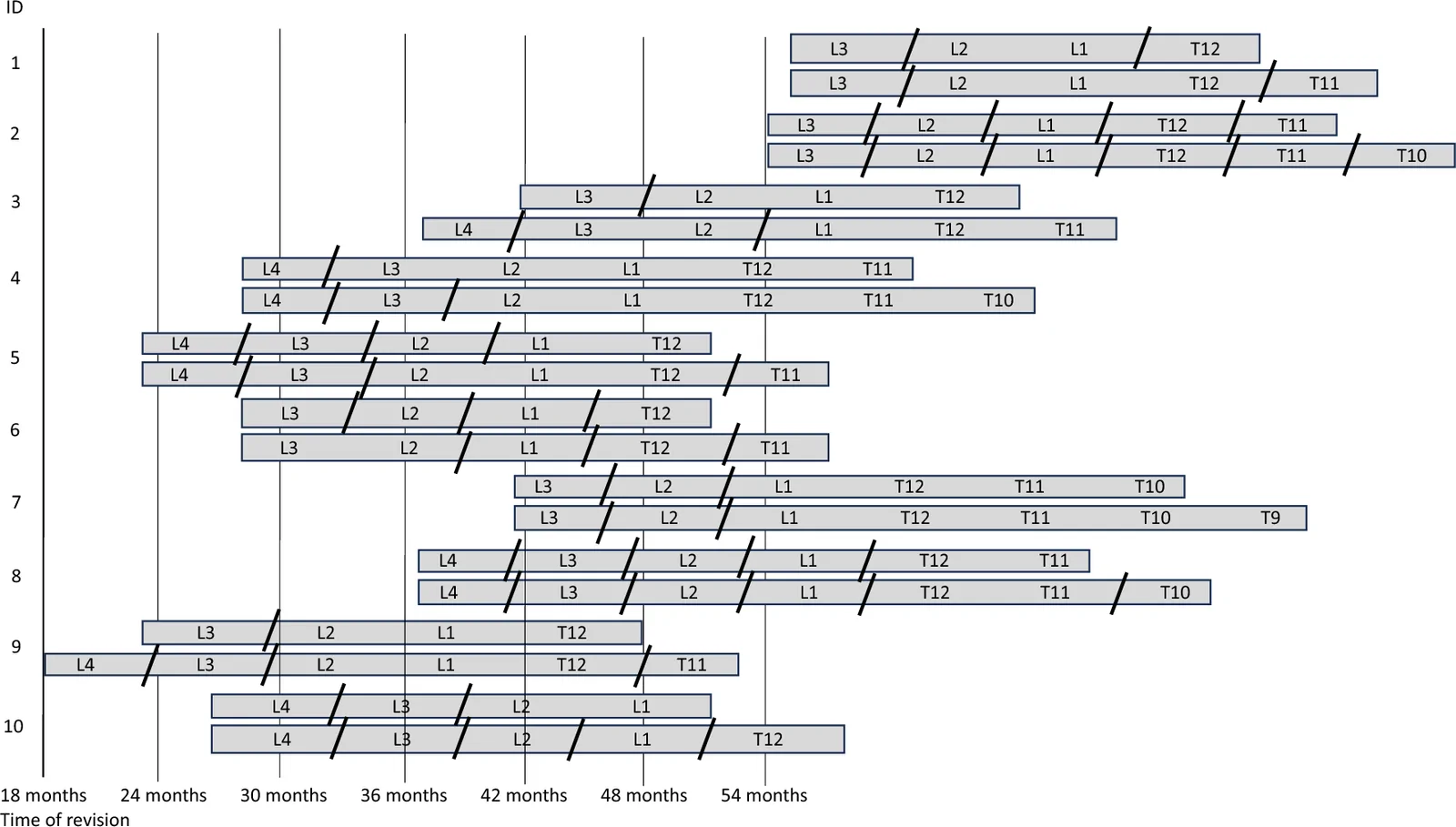
Tether breakages (TB) in relation to location and time of revision — In the illustration, the upper bar represents the anterior tether, and the lower bar represents the posterior tether for the respective patient

Data from 50 instrumented levels in ten patients were available **(**Table 1**)**. As shown in Fig. 2, the anterior and posterior tethers were not equally fixed along all the segments, resulting in 50 segments for the posterior and 38 segments for the anterior tethers. The mean age at index surgery was 15.1 ± 2.5 years. The study included nine females (90%) and one male (10%). The mean Risser stage was 3 with a standard deviation of 1.9 and a range of 0–5. The mean Sanders stage was 6.1, with a standard deviation of 1.5. The upper instrumented vertebra ranged from T9 to T11, and the lowest instrumented vertebra was L3 or L4. In our cohort, four of ten patients underwent bilateral VBT for double curves. Three had only the TL tether revised; one had both revised. The thoracic tether was excluded from analysis.

**Table 1:** Summary of the baseline, preoperative, postoperative data as well as tether breakage analysis. Pre- and postoperative data and average time to revision surgery are expressed as mean ± standard deviation. The periapical segments were defined as the segments above and below the apex

Baseline data		
*n*		10
Gender	female male	90% 10%
**Preoperative data**		
Age (years)		15.1 ± 2.5
Risser stage		3 ± 1.9
Sanders stage		6.1 ± 1.5
Preoperative	Cobb thoracic (°)	44.3 ± 15.8
	Cobb thoracic bend (°)	24.7 ± 12.2
	Cobb lumbar (°)	57 ± 7.8
	Cobb lumbar bend (°)	16.9 ± 8.3
**Postoperative data**		
1^st^ erect	Cobb thoracic (°)	21.5 ± 9.7
	Cobb lumbar (°)	14.3 ± 6.4
12-month post-op	Cobb thoracic (°)	27.9 ± 8
	Cobb lumbar (°)	29 ± 7.8
24-month post-op	Cobb thoracic (°)	31.9 ± 10
	Cobb lumbar (°)	33.6 ± 9
**Tether breakages**		
Average time to detection of first tether breakage (months)		14.55 ± 9.91
Average time to revision surgery (months)		34.8 ± 12.5
Radiographically suspected tether breakages		19 segments
Confirmed tether breakages		35 segments
Location of tether breakage	Posterior instrumentation	Proximal segment: 7/10 Periapical segments: 5/10 Distal segment: 9/10
	Anterior instrumentation	Proximal segment: 3/10 Periapical segments: 4/10 Distal segment: 10/10

The mean preoperative thoracic Cobb angle was 44.3° ± 15.8°, while the mean lumbar angle was 57° ± 7.8°. On preoperative bending radiographs, the mean thoracic curvature was 25°, and the mean lumbar curvature was 17°. Postoperatively, at first erect, the mean thoracic Cobb angle was 21.5° ± 9.8°, and the mean lumbar Cobb angle was 14.3° ± 6.4°. An increase in mean Cobb angles was noted at 12 months postoperatively. The mean thoracic Cobb angle was 27.9° ± 8°, and the mean lumbar Cobb angle was 29° ± 7.8°. At 24 months postoperatively, a further increase in the mean Cobb angle was reported. The mean thoracic Cobb angle was 31.9° ± 10°, and the mean lumbar Cobb angle was 33.6° ± 9°. Table 2 shows the thoracic and lumbar Cobb angles for each patient at the first erect position and before revision surgery.

**Table 2:** Thoracic and lumbar Cobb angles at 1^st^ erect and before revision for each patient

Patient ID	1st erect		Before revision surgery	
	Cobb thoracic (°)	Cobb lumbar (°)	Cobb thoracic (°)	Cobb lumbar (°)
**1**	38	23	37	52
**2**	29	22	28	42
**3**	24	8	50	36*
**4**	17	19	22	40
**5**	28	21	37	40
**6**	16	10	38	40
**7**	10	13	18	41
**8**	3	3	27	35 “
**9**	23	10	37	42
**10**	27	14	50	42

^*^Bilateral Revision; “severe coronal decompensation

### Analysis of time and location of tether breakages (TB)

The mean time to the first detection of tether breakage was 15 months, ranging from six weeks to 36 months. Revision surgery was performed on average 35 months after the index surgery with a range of 18 to 55 months. **(**Tables 1 and 3**)** However, no breakage pattern in relation to time could be observed. **(**Fig. 2**)** Patients with four or more breakages were observed among those who underwent revision surgery 24 to 30 months after their index surgery. Conversely, patients with three or fewer ruptures were observed among those who underwent revision surgery after 36 to 64 months.

**Table 3:** Tether breakages (TB) in relation to instrumentation levels, location of tether breakage, and time to revision (months)

Patient ID	Posterior instrumentation	Anterior instrumentation	Time to revision (months)	Location of tether breakage	
**1**	T11-L3	T12-L3	55	ant	T12-L1 L2-3
				post	T11-12 L2-3
**2**	T10-L3	T11-L3	54	ant	T11-12 T12-L1 L1-2 L2-3
				post	T10-11 T11-12 T12-L1 L1-2 L2-3
**3**	T11-L4	T12-L3	37	ant	L2-L3
				post	L1-2 L3-4
**4**	T10-L4	T11-L4	28	ant	L3-4
				post	L2-3 L3-4
**5**	T11-L4	T12-L4	23	ant	L1-2 L2-3 L3-4
				post	T11-12 L2-3 L3-4
**6**	T11-L3	T12-L3	28	ant	T12-L1 L1-2 L2-3
				post	T11-12 T12-L1 L1-2
**7**	T9-L3	T10-L3	41	ant	L1-2 L2-3
				post	L1-2 L2-3
**8**	T10-L4	T11-L4	37	ant	T12-L1 L1-2 L2-3 L3-4
				post	T10-11 T12-L1 L1-2 L2-3 L3-4
**9**	T11-L4	T12-L3	18	ant	L2-3
				post	T11-12 L2-3 L3-4
**10**	T12-L4	L1-L4	27	ant	L2-3 L3-4
				post	T12-L1 L1-2 L2-3 L3-4

Pictures of explanted tethers showed breakage at disc-level, not at the screw–tether interface. Furthermore, tether breakage was confirmed at 35 levels of which only 19 levels (54%) were suspected to be broken. At 19 segments, both tethers were broken, 13 of these were radiographically suspected (68%). The posterior tether broke in 31 out of 50 segments (62%). Seven of ten patients showed a breakage of the proximal segment, five a periapical one, and nine a breakage of the distal segment. The anterior tether broke in 23 out of 38 segments (61%). Three patients had a proximal breakage, four had a periapical breakage, and ten had a distal tether breakage. The L3/L4 tether was broken in six out of six patients (in five patients with double-row tethers and one patient with single tether). The L2/3 segment was also broken in all cases. The L1/2 segment broke in four cases. The L2/3 segment was broken in all four patients with L3 as LIV, and three of these patients also had a breakage at the L1/2 segment.

## Discussion

**Fig. 3:**
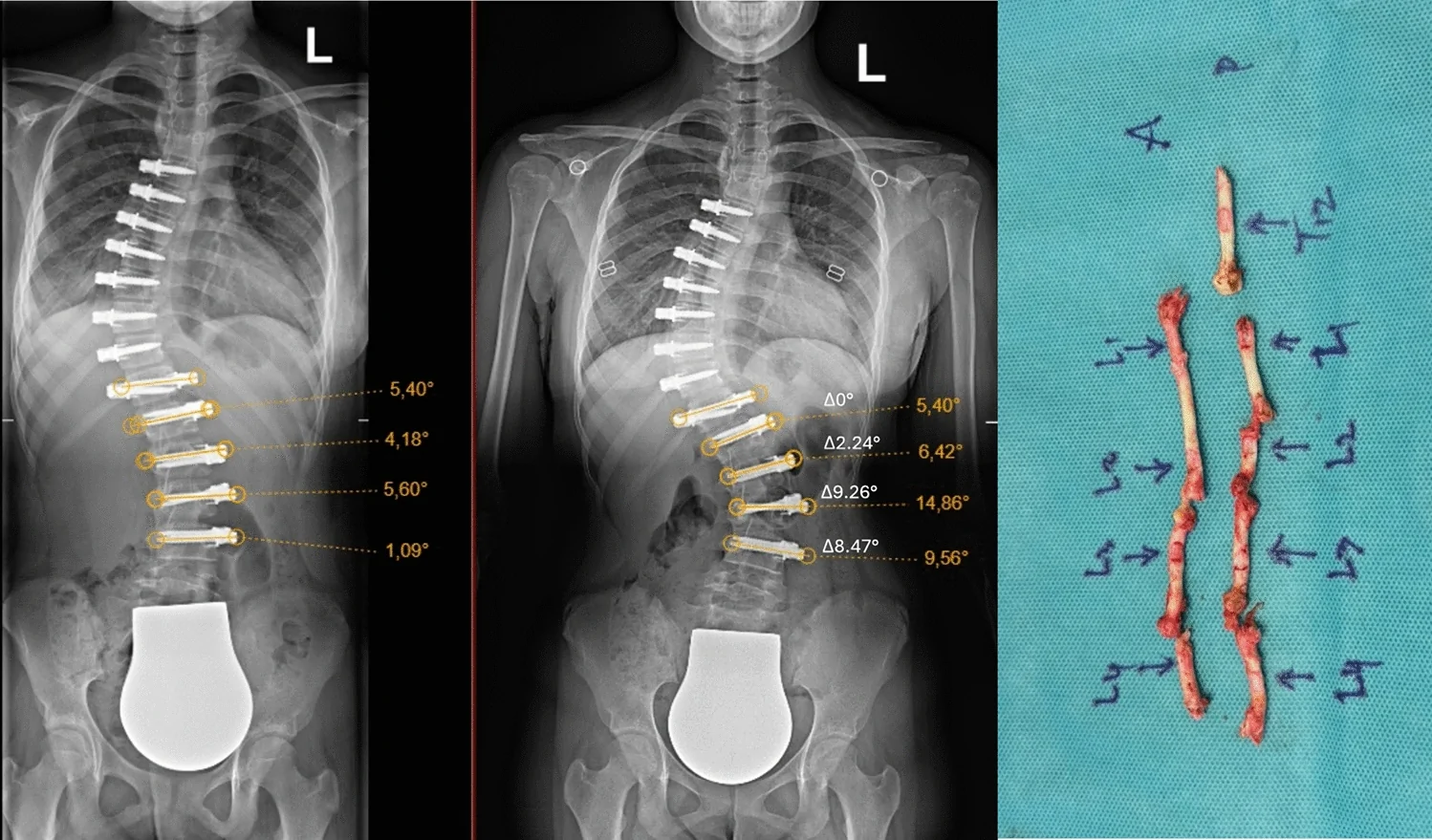
Example case of a 11 + 7-year-old girl who has had double VBT at Risser 0 and Sanders Stage 3. 1st erect and 27 months post-op radiographs suspected tether breakages at L2/3 and L3/4. Intra-op explantation additionally identified breakages of the posterior tether at T12/L1 and L1/2

**Fig. 4:**
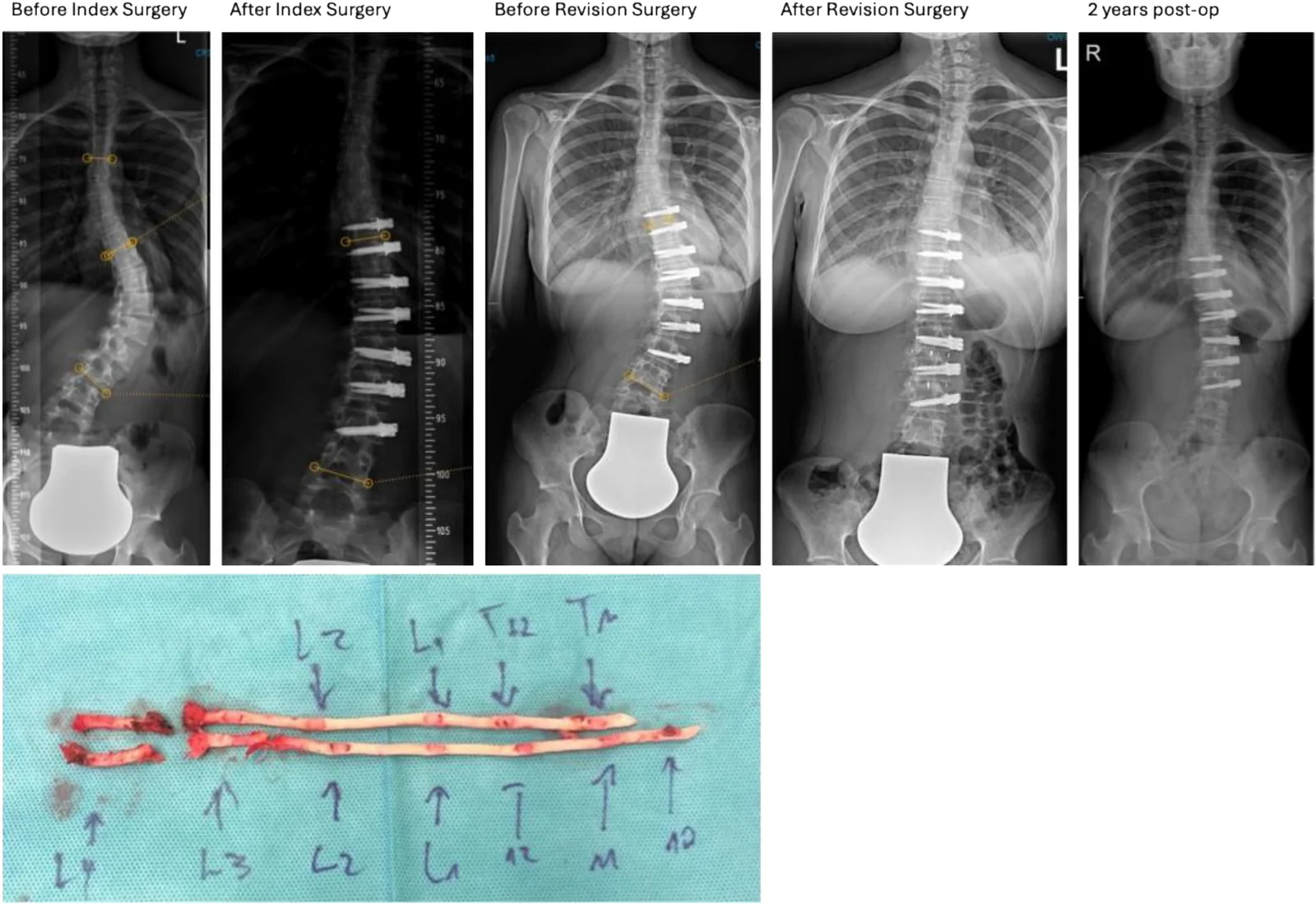
Example case of a 16 + 9-year-old female patient who has had double-row VBT at Risser 4 and Sanders 8. The follow-up examinations revealed a continuous progression of the lumbar Cobb angle: 19° at 1st erect, 26° 12 months post-op, and 40° before the revision surgery. Revision surgery was performed 28 months after index surgery. The anterior tether broke between L3/4 and the posterior tether between L2/3 and L3/4

The main finding of this study was that the tethers broke most often in the distal segment. As shown in Figs. 2, 3, and 4, this suggests that lumbar tethers are prone to breaking. This finding is consistent with those of other clinical studies. In their follow-up study of at least five years, Yang et al. described that the majority of lumbar and thoracic tether breakages occur at or distal to the apex [17]. Another study found that tethers most often break at the disc-level, near the lowest instrumented vertebra [15].

Additionally, the data showed that tether breakage occurred more frequently in the lumbar spine than in the thoracic spine. This finding from this small clinical study is consistent with previous studies that have reported higher breakage rates for lumbar instrumentation compared to thoracic instrumentation [17–19]. Baroncini et al.’s study showed a 71% risk of tether breakage for lumbar VBT, compared to 29% for thoracic VBT [7].

The literature does not offer a single answer to the pooled occurrence of lumbar or distal tether breakages. First, biomechanical studies have already shown higher forces in the lumbar spine overall. Higher forces in the lumbar segments also mean that higher forces act on the tether. Additionally, a multi-body simulation study showed that lumbar tether forces are two to three times greater than thoracic tether forces during motion [11, 20].

Second, the lumbar range of motion (ROM) is greater than the thoracic ROM [21, 22]. A higher ROM means more compression and tension on the tether. Clinical studies have shown that fusing the apex of the spinal curvature and thus eliminating the ROM at the segment at risk for breakage reduce tether breakage rates [13]. A previous biomechanical study analyzed this approach from a biomechanical standpoint and found a reduction in tether forces in the fused and adjacent segments, explaining the clinically observed reduction of the breakage rate [12].

Mean time to first detection of tether breakage was 15 months (range 6 weeks–36 months); only one case occurred at 6 weeks. Additional breakages were often identified during follow-up before revision was indicated. Given the small sample, distinguishing acute from fatigue-related failures is challenging; microstructural studies are needed to clarify etiology.

Furthermore, only 54% of tether breakages were identified through radiography. Previous studies have shown that the > 5° rule has low sensitivity; only 56% of tether breakages that were later confirmed intraoperatively were detected on radiographs. Nevertheless, many tether breakages do not result in loss of correction [14]. In this retrospective study, however, radiographically suspected tether breakages often identified a segment in which both tethers were broken. Therefore, the > 5° rule may only be sufficient for identifying double tether breakages.

Anterior and posterior tethers were affected similarly (61% vs. 62%). Macrostructural analysis **(**Figs. 3 and 4**)** showed breakage at the disc-level, not at the screw–tether interface. Further biomechanical/material studies under cyclic loading (tension/compression) are needed to elucidate microstructural failure mechanisms. The goal is to refine tether design and microstructure to preserve flexibility while preventing breakage.

No clear pattern of timing was observed in our study. However, this finding aligns with those of other clinical studies, which suggest that age or skeletal maturity is not a risk factor for tether breakage. Both Baroncini et al. and Yang et al. found that age and skeletal maturity were not risk factors for the biomechanical complication of tether breakage [7, 17]. Tether breakage is still a multifactorial event that requires further clinical study [23, 24].

Today, radiography is the gold standard for follow-up diagnostics. Although CT scans may be more reliable, they have the disadvantage of increased radiation exposure [25]. The future goal should be to radiographically detect tether breakage with a sensitivity greater than 56% [14]. However, not every tether breakage results in revision surgery. Eaker et al. reported that 83% of patients with a tether breakage were considered clinically successful, with a major curve under 35 degrees [10]. The timing of tether breakage, curve magnitude, and skeletal immaturity play a critical role in the risk of curve progression [9]. Currently, tether breakage can only be reliably identified intraoperatively during revision surgery because the tether itself is radiolucent. Modifying the material and radiolucency of the tether could improve detection of tether breakage.

However, the study has the following limitations: it is an observational, retrospective analysis including only a small number of patients. The small number of patients is mainly because the study analyzes tether breakage after double-row thoracolumbar VBT. VBT remains a highly specific procedure, and revision surgeries for double-row TL tether breakage represent a small and rare subgroup. Furthermore, as previously mentioned, not all tether breakages necessitate revision surgery, and approximately, 83% of cases are considered successful even with a tether breakage [10]. Next, the authors performed a descriptive analysis of the broken tether cords based on the photographic documentation. No biomechanical analysis was performed. This study analyzed the tether breakage pattern on a macrostructural level. Further studies are needed to analyze breakage patterns at the microstructural level.

We did not quantify recommended or performed revisions because decisions were shared with patients and families. As a retrospective series limited to TL double‑row tethers that required revision, we did not evaluate overall tether‑breakage prevalence and no risk‑factor analysis was performed. While improved implant design and surgical technique have been associated with superior radiological outcomes and lower breakage rates, reported rates remain inconsistent [7, 10, 17, 26]. For TL curves, Tetreault et al. observed higher breakage in single‑tether constructs (60%) than double‑tether constructs (35%), suggesting a protective effect [9]. However, Shankar et al. found no significant difference (32% single vs. 30% double) [27]. Further studies are needed to better understand the effect of the use of double tethers.

Furthermore, our study population shows some heterogeneity regarding Risser and Sanders stages, with a mean Risser stage of 3 ± 1.9 and a mean Sanders stage of 6.1 ± 1.5. During the initial years of VBT, there were no clear indication criteria for TL curves. The implant was approved for both immature and mature patients. This study includes a small number of mature patients with flexible lumbar curves, who were believed to benefit from Wolff’s law by disc remodeling.

Skeletal maturity influences outcomes: Alanay et al. [28] reported higher mechanical complications and overcorrection at lower Sanders stages — particularly Stage 2 — while Stages 3–5 had lower risk. Tetreault et al. [9] found breakage rates were not significantly associated with Sanders or Risser stage, but skeletally immature patients with larger curves at breakage had a higher likelihood of requiring revision. Meyer et al. showed VBT can achieve meaningful correction even with limited remaining growth [29]. In mature patients, tether breakage was relatively common (41%), yet revisions were rare (2%). Collectively, these data underscore the importance of careful patient selection and optimal timing of VBT.

We acknowledge that data from this small population of patients who underwent TL VBT after skeletal maturity are still being analyzed for mid- and long-term outcomes. For now, we have stopped applying VBT to mature patients, and we do not recommend that other surgeons do so until more data have been analyzed. In our practice, immature patients (Sanders 3 to 5) with flexible curves below 60° are now considered as good candidates for TL VBT. Hybrid options consisting of a combination of apical fusion and VBT are considered for patients with more rigid curves or for more mature patients (up to Sanders 7). Preliminary clinical and biomechanical data regarding this technique have been published [12, 13]. The data on the two-year follow-up clinical outcomes are currently under peer review.

In summary, this is, to our knowledge, the first study to analyze actual tether breakages in double-row TL VBT. The patient population is highly homogeneous, and all cases were analyzed consecutively. Thus, this study offers valuable insights into the ongoing debate about the pathomechanism of TL tether breakages.

## Conclusion

Consecutively explanted TL tethers did not show a clear pattern of breakage. The distal segments were affected in almost all cases. Anterior and posterior implants were equally affected, and no clear pattern of breakage in relation to the time the tether was standing was observed. Tether breakage remains a multifactorial event.

## Data Availability

The data generated and analyzed during the current study are available from the corresponding author on reasonable request.
